# Red mangrove life history variables along latitudinal and anthropogenic stress gradients

**DOI:** 10.1002/ece3.1095

**Published:** 2014-05-13

**Authors:** C Edward Proffitt, Steven Travis

**Affiliations:** 1 Department of Biological Sciences, Florida Atlantic University, c/o Harbor Branch Oceanographic InstitutionFt. Pierce, Florida, 34946; 2 Department of Biology, University of New EnglandBiddeford, Maine

**Keywords:** Cold stress, global change, mangrove, mutation, outcrossing, reproductive output

## Abstract

Mangroves migrate northward in Florida and colonize marshes historically dominated by salt marsh species. In theory, this migration should be facilitated by greater numbers of propagules stemming from increased reproductive activity and greater genetic variability caused by outcrossing. We aimed to determine if stand reproduction and % outcrossing were affected by cold stress (stress increases with latitude), anthropogenic stress (human population density as a proxy), and years since a major hurricane. Further, we wished to determine if mutation rate varied with the stressors and if that affected stand reproduction. Both coasts of Florida from the southern Florida Keys to Tampa Bay on the Gulf of Mexico coast, and Merritt Island on the Atlantic coast. We conducted field surveys of frequency of reproducing trees (104,211 trees surveyed in 102 forested stands), incidence of trees showing albinism in propagules, and% outcrossing estimated from the ratio of albino:normal propagules. Structural equation modeling (SEM) was used to test a conceptual model that served as a multivariate hypothesis. Reproductive frequencies varied by site and increased with latitude although more strongly on the Gulf coast. Our SEM results indicate that outcrossing increases in this predominately selfing species under conditions of cold and anthropogenic stress, and that this increases reproductive output in the population. Further, we find that increased mutation rates suppress stand reproductive output but there is no significant relationship between outcrossing and mutation rate. Tree size responded to stressors but did not affect stand reproduction. Reproduction increased with years since major hurricane. Potential for colonization of northern Florida salt marshes by mangroves is enhanced by increased reproductive rates that provides more propagules and outcrossing that should enhance genetic variation thereby promoting adaptation to novel environmental conditions. Natural (cold) stress reduced mutation rate and increased stand reproductive output but anthropogenic stress did the opposite.

## Introduction

Temperatures are projected to rise 0.5 to 2 C by 2035 (Stocker et al. 2013) which will affect the physiological ecology of and interactions among many species. Changes in winter cold extremes will become less frequent which will lead to colonization of temperate sites by some tropical and subtropical species. Plant phenology will be affected by temperatures and changes in carbon dioxide concentrations (see review by Hughes 2000) as will other aspects of life history and interactions with pollinators, parasites, and herbivores. For example, onset of flowering has advanced 3-8 days over a 150 year record of the locust tree (Walkovszky 1998). It is likely that there will be critical differences among major groupings of plants (e.g., herbaceous plants vs. trees) in their responses to global change. For trees, certain plant traits, pollen dispersal, and outcrossing may allow the diversity necessary for some populations to adapt to novel environments (Hamrick 2004).

*Rhizophora mangle* L., the red mangrove, is a salt-tolerant tree that dominates intertidal zones on low wave energy shorelines in the New World tropics and subtropics. It is considered to be a habitat-forming foundation species and has profound effects on fishery and avian productivity, storm surge abatement, and reduction in erosion (IUCN 2007). *Rhizophora mangle* has perfect flowers and so all individuals are capable of bearing offspring. It has been reported to be a highly selfing species, with the anthers usually dehiscing before the flower bud opens (Lowenfeld and Klekowski 1992; Klekowski et al. 1994a,b1994b,c1994c; Proffitt and Travis 2005). For selfing populations, the primary mode of dispersal are the propagules produced by self-fertilization.

In Florida, populations of *R. mangle* and other mangroves exist in a realm of multiple stressors, including natural sources such as the cold gradient associated with increasing latitude and anthropogenic sources such as runoff laden with nutrients, heavy metals, and pesticides. The effects of these multiple stressors acting in concert are poorly understood (Proffitt and Travis 2005) but may be central to understanding the northward migration of mangroves with warming of the climate. Both the rate of colonization of novel habitat and stress-related differences in mangrove tree architecture will have important ecological consequences to plant reproductive output, interactions with the largely herbaceous salt marsh species, and use by fauna.

We focus here on the effects of climate and anthropogenic stressors on four aspects of *R. mangle* life history: stand reproductive output, outcrossing rate, mean tree size, and albino mutation rate. In Florida, populations of *R. mangle* and other mangroves advance northward during a series of warm winters, and are killed back by hard or extended periods of freezing temperatures (Cavanaugh et al. 2014). As global climate changes, it is likely that mangroves will invade and eventually dominate temperate salt marshes in northern Florida and the northern Gulf of Mexico. Plants colonizing regions with colder temperatures are entering novel selective landscapes. In theory, elevated levels of outcrossing should increase genetic diversity and facilitate adaption to the new selection regime, and thus individuals that outcross in any environment or mechanisms that induce outcrossing might be favored in northern climes. Also, stress, including thermal stress, can increase mutation rates, that may enhance genetic diversity upon which selection can work to produce locally adapted sets of genotypes. Stress may also affect individual tree, and thus entire stand, reproductive output and reproduction is key to colonizing novel temperate habitats as well as more recolonizing sites disturbed by hurricanes or human impact in the tropics and subtropics. Reproductive output may also be less in northern climes where trees are typically smaller.

**Figure 1:**
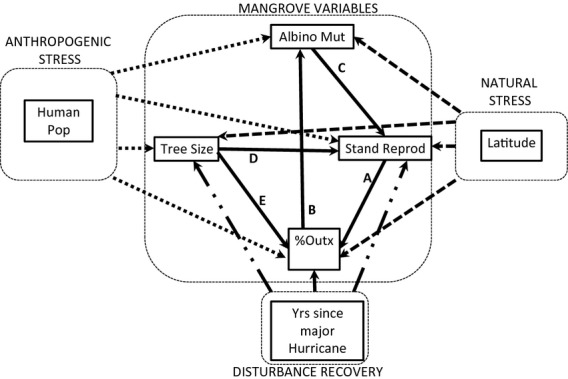
The full conceptual model linking mangrove size and life history variables to anthropogenic stress (human population size), natural stress (latitude), and years of recovery from disturbance by major hurricanes which is hypothesized to influence several mangrove variables. Solid lines indicate the mangrove variables. Long-dash lines indicate the effects of natural cold and freeze stress. Short dashes indicate anthropogenic stress. Hurricane disturbance is indicated by a dash-dot line. EXPLANATION OF LINKAGES AMONG THE MANGROVE VARIABLES. (A) As the density of reproducing trees km^−1^ increases the likelihood of pollen transfer among trees increases. (B) Greater outcrossing will mask to some degree the frequency of albinism that depends on self fertilization in a heterozygotic tree. (C) Higher mutation rate can be used as one measure of sub-lethal stress in the population. Stress (the indicator variable mutation rate) can reduce frequency of reproduction. (D) Larger trees will be spaced further apart thus reducing the number of reproducing trees km^−1^. (E) Larger trees produce more flowers/tree which may increase the incidence of outcrossing. Variables are: Albino Mut = frequency of occurrence of reproducing trees heterozygotic for albinism, Stand Reprod = number of reproducing trees km^−1^, % Outx = the mean percent outcrossing in a stand, and Tree Size = tree trunk DBH. A direct link between outcrossing and reproduction was also hypothesized based on genetic theory.

We used the theoretical relationships described above to develop a conceptual path model as a multivariate hypothesis (Fig. 1). This multivariate hypothesis was tested by structural equation modeling (SEM) analysis of observational data collected in the field. We consider this as an exploratory SEM and offer the results as testable predictions for future research.

## Methods

### The plant variables

Stand reproductive, mutation, and outcrossing rates can be readily measured in the field for *R. mangle* by simple shoreline surveys of reproducing trees (Lowenfeld and Klekowski 1992; Klekowski et al. 1994a,b1994b,c1994c; Proffitt and Travis 2005). *Rhizophora mangle* is viviparous, and seedlings grow to large size, sometimes exceeding 30 cm in length, while still attached to the maternal tree. Thus, the rate of mutation to the albino phenotype, which is otherwise lethal, is directly observable because propagules on parent trees are nourished by stored maternal reserves, and heterozygotic trees can be visually distinguished from normal trees when they are reproducing. The species is often dominant at the interface with open water, which allows convenient surveying of the population by boat or walking. Normal propagules are green; albino propagules are yellow, pink, or white, and can be easily distinguished from normal propagules once they are of mature size. Mutation rates are estimated from the frequency of occurrence of heterozygotic trees (those producing both normal and albino propagules) in the entire population of reproducing trees (Lowenfeld and Klekowski 1992; Klekowski et al. 1994a,b1994b,c1994c; Proffitt and Travis 2005). Here, we use the incidence of albinism as an estimate of overall mutation rates throughout the genome. Rather than calculating mutation rates per unit of genetic information, as is traditional, we report the frequency of occurrence of trees producing albino propagules for each stand of trees.

The degree of outcrossing for a heterozygotic tree can be estimated as the magnitude of the deviation from a 3:1 Mendelian ratio of normal to albino propagules expected with self- fertilization (Klekowski et al. 1994c). We use the proportion of heterozygotic trees in a stand that significantly deviate from this ratio as a measure of the outcrossing rate of that stand (Proffitt and Travis 2005).

Our field surveys closely followed the protocol of (Proffitt and Travis 2005). Depending on site conditions, all reproducing *R. mangle* that can be seen are usually counted from a boat, or sometimes by walking the shoreline. This generally includes trees within 2–5 m of the edge of open water. Our field observations and work by Devlin (2004) indicate that most reproduction in many if not most red mangrove population is by individuals growing closest to open water. For tree size, we measured the diameter at breast height (DBH) and height of each heterozygotic tree.

### Environmental and human population variables

Latitude was measured for each stand in the field using hand-held GPS. Numbers for human population in centers nearest a site were taken from the 2008 U.S. Census.

### Statistical analyses

We tested the explanatory power of the conceptual meta-model in Fig. 1 using structural equation modeling (SEM) with manifest variables (Shipley 2000; Grace 2006). SEM allows multivariate hypotheses to be tested, and permits assessment of the relative importance of both direct and indirect effects. An entire model is tested for fit to the data by comparing the covariance structure of the data with the expected covariance structure implied by the proposed set of pathways in the hypothetical model (Shipley 2000; Grace 2006). Data were log_e_ transformed, or in the case of reproductive output (counts of reproducing trees) square root transformed, prior to analyses. All analyses were done in Mplus.

In Fig. 1, the primary response variable of interest, forested stand reproduction was measured as the number of reproducing trees km^−1^ of red mangrove dominated shoreline. Increasing latitude was used as measure of cold stress, and human population densities the proxy for anthropogenic stress. Greater human population density captures “urban runoff” pollutants, but misses some agricultural pollutants in more rural watersheds. Urban areas are known to be important sources of PAHs and other contaminants (Bombai and Hernandez 1991; Ngabe et al. 2000), which are known to be toxic to some estuarine species (Coull and Chandler 1992) and their toxicity is increased by photoactivation after exposure to light (Arfsten et al. 1996). Runoff from urban sediments can have sublethal effects on reproduction in some species (Bejarano et al. 2004). Further, in much of south Florida agricultural contaminants flow into canals or rivers that then pass through urban centers, thereby being “counted” along with the “human population density” of that location. Tree size, mutation rate (incidence of albinism), and outcrossing rates were inherently biological factors. We also tested several other hierarchical models nested within the full model shown in Fig. 1: one without tree size, one without anthropogenic stress, and one without both tree size and anthropogenic stress (On-line Supplementary Information Table S1). Model comparisons using AIC indicated that the full model had the greatest support (On-line Supplementary Information model probability = 0.64, Table S1), and hence, only results for this model are presented.

We tested via SEM the multivariate hypothesis that stand reproductive output is a function of biological (tree size, outcrossing rate, and mutation rate) and environmental (cold stress and anthropogenic stress) variables that act in direct and indirect paths as postulated in Fig. 1.

## Results

### Relationships among biological and stress

**Figure 2:**
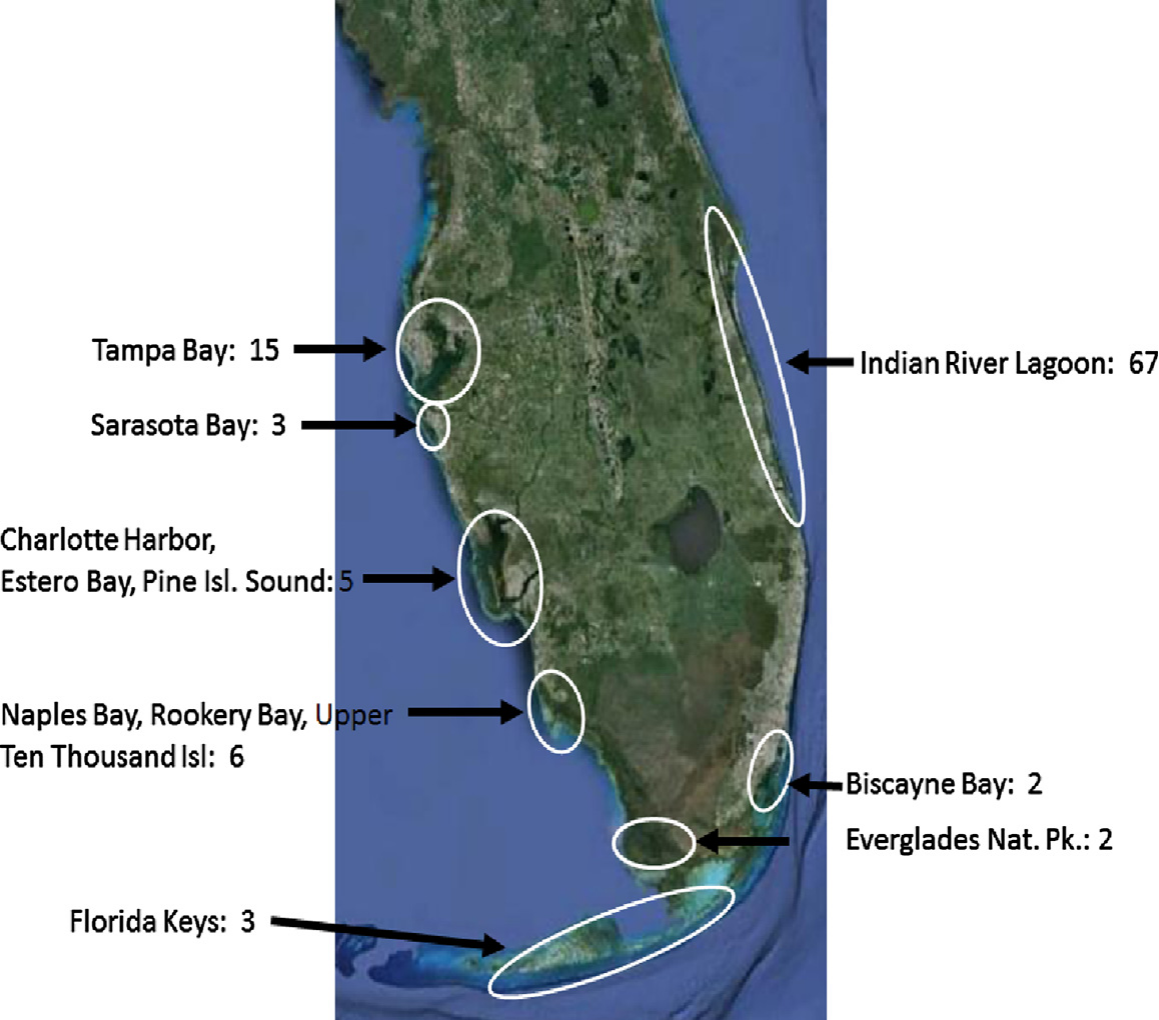
The locations of Florida surveyed in the study are indicated by the ovals and explanatory text. Numbers of individual forested stands surveyed in each location are given.

Our surveys from 2001–2012 included 102 forested stands ranging from Tampa Bay on the Gulf of Mexico coast to Merritt Island in the Indian River Lagoon on the Atlantic coast. The surveys covered a range from 24 to 28 degrees N latitude. We counted 104,211 total reproducing trees, and found 431 heterozygotic trees segregating for albinism mutations. There was a statewide median frequency of heterozygotic trees segregating for albinism of 4.36 heterozygotes per 1000 reproducing trees. Mean shoreline surveyed per site was 2.4 km ± 3.01 (SD) Fig. 2.

**Figure 3:**
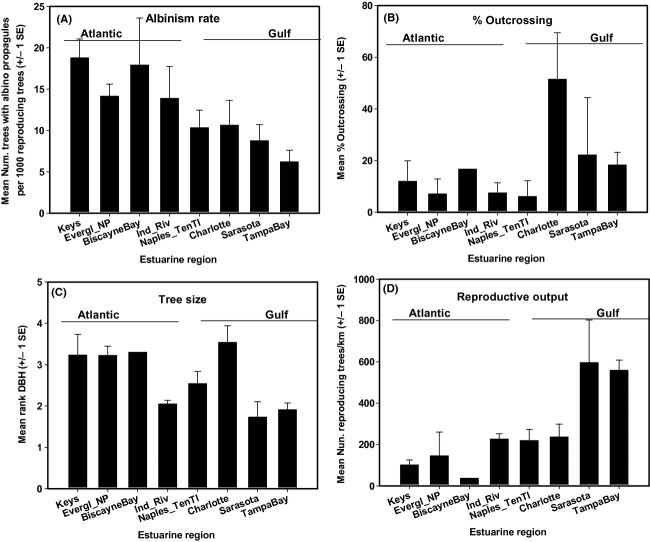
(A) Numbers of heterozygotic red mangrove trees segregating for albinism, (B) Percent outcrossing, (C) Tree size, and (D) total reproducing trees per km among south Florida's estuaries. Estuaries on Atlantic and Gulf coasts are indicated.

*Rhizophora mangle* reproduction, outcrossing, and frequency of occurrence of trees showing albinism in propagules varied substantially among and within estuaries and between Gulf and Atlantic coasts (Fig. 3). The pattern was one of greater mutation rates (estimated by frequency of heterozygotic trees producing albino propagules) on the southeast coast, and greater forest reproductive output and outcrossing in many estuaries of the Gulf coast. The range in stand reproductive output was striking at 4–878 reproducing trees km ^−1^. Some forested stands in the Indian River Lagoon have been surveyed repeatedly in order to ascertain cycles in reproductive output and to determine if larger patterns seen are stable. Wildcat Cove typically has low reproductive output (47.3 ± 12.5 SE, *n* = 10 surveys), while in comparison Olso South has much greater reproduction (450.2 ± 32.1, *n* = 10 surveys) although tree sizes and densities are similar. For sites surveyed more than once, means of variables were used in SEM.

### Structural equation modeling analysis

**Figure 4:**
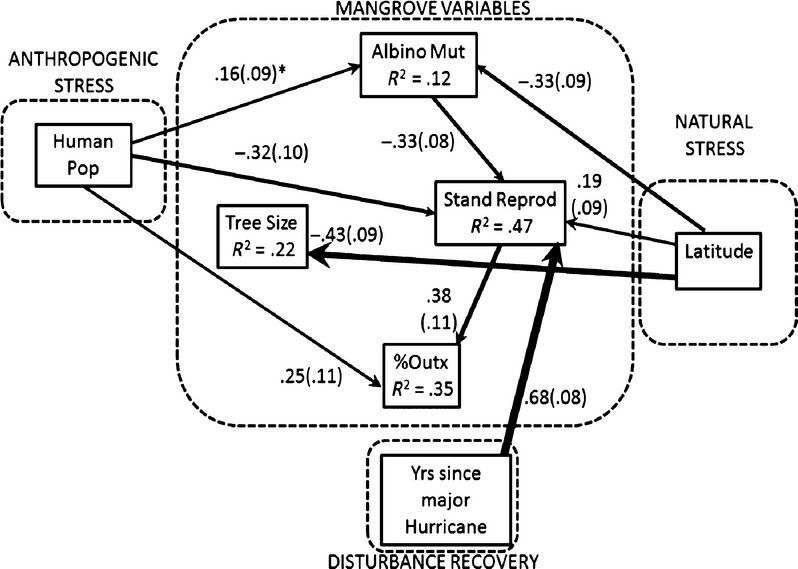
The final SEM linking mangrove size and life history variables to anthropogenic stress (proxy human population size), natural stress (colder winters and increasing likelihood of frost with increasing latitude), and the number of years of recovery from disturbance by major hurricanes which is hypothesized to influence several mangrove variables. *n* = 102 cases. MODEL FIT INDICES: Chi Sq = 3.65, df = 3, *P* = 0.302; CFI = 0.994, TLI = 0.963, RMSEA = 0.046, *P* = 0.409, AIC = 848.8, num. free parameters = 23. Standardized path coefficients are shown and values in parentheses are standard errors. *Indicates only marginally significant (*P* = 0.09).

Mangrove population, mutation rate, degree of outcrossing, and reproductive output were affected by cold stress and freezes associated with higher latitudes and anthropogenic factors associated with human population density (possibly pollutants in runoff, although these were not quantified), and the years of recovery from major hurricane disturbance (Fig. 4). Overall, the model as a multivariate hypothesis fit the data better than any other model tested (Fig. 4 and Appendix Table S1). The frequency of trees producing propagules (*R*^2^ = 0.47) increased with the number of years since last major hurricane. The degree of estuary urbanization, as evidenced by the density of human inhabitants, had a negative effect on reproductive frequency. Higher mutation rates, as indicated by the frequency of trees producing albino propagules, had a negative effect on frequency of reproduction.

Tree size (*R*^2^ = 0.22) decreased with increasing latitude because of cold and possibly frequency of freezes. Tree size, however, did not affect any of the other mangrove variables (Fig. 4).

The percent outcrossing (*R*^2^ = 0.35) was greater at colder latitudes via indirect pathways through stand reproduction and mutation rate (total standardized effect size through these pathways = 0.11, the product of the standardized path coefficients). Increased reproductive output (density of reproducing trees km^−1^) also led to greater percent outcrossing, and there was a positive indirect effect (0.26) on% outcrossing of years since major hurricane through the stand reproduction path (Fig. 4). Higher mutation rates decreased% outcrossing indirectly through a negative effect on stand reproduction (−0.13). Mutation rate (*R*^2^ = 0.12) decreased with latitude but was not affected by% outcrossing as had been hypothesized. Several biological variables were also affected by proximity to human population centers. The rate of mutations was greater, stand reproduction decreased (direct effect = −0.32, indirect effect = 0.05) for a total effect of −0.37), and% outcrossing increased (direct effect = 0.25, offsetting indirect effects = −0.14 for a total effect of 0.11) (Fig. 4).

## Discussion

Two life-history traits, reproductive effort (numbers of reproducing trees km^−1^) and % outcrossing that may be important for *R. mangle* populations successfully invading north Florida salt marsh areas as global climate changes were increased by the cold stress of higher latitudes. The number of years since a major hurricane hit or passed near a stand had the greatest (positive) effect on stand reproductive output (Fig. 4). Once recovery from hurricane disturbance was accounted for in the SEM, the effects of natural (cold) and anthropogenic stress became clearer. Cold stress had both direct and indirect (by reducing mutation rates) positive effects on stand reproduction. Cold stress also had an indirect positive effect on percent outcrossing through reproduction and mutation pathways. The colder winters with presumably shorter growing season and greater probability of exposure to frost at higher latitudes led to reduced albino mutation rates. The mechanism for this is not clear, but could be related to slower metabolic rates during colder months reducing the likelihood of mutations occurring. Alternatively, cold stress may select against seedlings that are heterozygotic for albinism if normal alleles are not completely dominant over albino alleles.

Cold stress not only increased the tendency of trees to flower (reproductive potential), but also to produce outcrossed propagules (perhaps increased seedling vitality). It should be noted, however, that the positive effect of cold stress will be partially offset by its tendency to limit lifespans, thus preventing trees from growing to large sizes. We suggest as a hypothesis for future study, that greater numbers of reproducing trees km^−1^ and higher outcrossing rate acting in concert may produce a swarm of new genotypes in northern climes that may facilitate adaption to the colder regions. These results are consistent with theory and data indicating that predictable stress can be a selection force (Via et al. 1995), and that stress can affect the timing or rate of reproduction (Stanton et al. 2004).

We did not survey sites at the extreme end of the *R. mangle* range because there were insufficient numbers of individuals to obtain good estimates of either reproductive output or mutation rates. Near the end of the latitudinal range, tree density will be so low that % outcrossing will be negligible because of the absence of nearby neighbors. Our results, however, suggest that if selection acts on the seedlings as described above, then the young trees produced by the surviving seedlings, could produce cohorts of well-adapted seedlings via self-fertilization. Thus, cycling from periods of selfing in young sparsely populated stands to outcrossing in more established, denser stands may be a driving mechanism that promotes northward expansion of *R. mangle* as it adapts to climatic stress.

This hypothesized model for *R. mangle* response to colder temperatures leads to testable predictions for future work. We predict from our results that, except at the extreme end of the range where abundances are very low, northern populations of *R. mangle* will have greater genetic variation than populations in more central parts of the range because of increased outcrossing. This is in contrast to the common belief that highest genetic diversity occurs in the center of a species' range; and, in this instance may in part be a function of the high selfing rates in *R. mangle* that may reduce genetic diversity in populations near the center of the range. Another prediction is that because the life history parameters we noted may facilitate adaptation to local environmental conditions, propagules in northern latitudes will be on the average more tolerant of freezes than those produced in southern Florida latitudes. There is literature suggesting latitudinal differences in cold tolerance of various mangrove species, but whether those differences were acclimation or heritable is not known (Sherrod et al. 1986; Krauss et al. 2008).

Our data suggest that higher mutation rates can reduce the frequency of reproduction in red mangrove populations. The reduced ability of mangrove trees to successfully reproduce when carrying a heavy mutational load is perhaps no surprise. What is perhaps more interesting is that cold stress per se appeared to lead to a reduced incidence of mutations. Mutational load produces adverse effects in experimental radish populations (Roles and Conner 2008). Radish populations in the greenhouse suffered up to 11% reduced fitness after 9–10 generations because of accumulated mutations when living in harsh environmental conditions, and field populations showed a similar non-significant, but highly variable, trend (Roles and Conner 2008). Thus, the mutation-fitness literature and our *R. mangle* data and analyses, are consistent with the interpretation that mutational load can occur in this habitat-forming ecological foundation species and that differential tolerance to cold or frost may be a mechanism for reducing this load and increasing fitness. Alternatively, reduced metabolic rates associated with lower temperatures might lead to a lower observed mutation rate.

Stress from anthropogenic sources (primarily point and non-point source runoff of contaminants) increased mutation rates and outcrossing rates, and decreased reproductive output (Fig. 4). Our previous work showed a positive relationship between contamination history by major chemical spills and industrial discharges and albino mutation rates in Florida red mangroves but did not find evidence for non-point source pollution on mutation rates in Tampa Bay (Proffitt and Travis 2005). The present study, thus, constitutes the first link between non-point source pollution and mutation rates. Because we use a proxy measure (human population density) rather than actual measurements of pollutants, this finding needs to be verified by further research. By increasing mutation rates and reducing reproductive output, anthropogenic effects are tending to counteract the positive response by *R. mangle* to cold stress described above. However, anthropogenic effects are also increasing outcrossing rates which may lead to a positive effect on genetic diversity and local adaptation, so the overall effects of human influences on the system are somewhat unclear and warrants further research.

In summary, evidence is mounting that adaption by red mangroves to changing climate can be affected by mutation and outcrossing rates along with reproductive output (this study) and maternal genotype x environment interactions (Proffitt and Travis 2010). Several findings from these two studies may influence the rate and success of *R. mangle* invasion of northern Florida salt marshes. Cold stress stimulates reproduction and reduces mutation rate, both leading to greater outcrossing. More outcrossing should lead to increased genetic variation, which provides more variation for genotype X environment interactions. If locally adapted genotypes are selected for, then the propensity for greater selfing at low population densities near the edge of the range could allow for locally adapted cohorts of seedlings to be produced. This could enhance the rate of colonization of environmentally marginal habitats.

## Supporting Information

**Table S1**. The four SEM models tested.
