# Supplementary material for: Red mangrove life history variables along latitudinal and anthropogenic stress gradients
Source: Ecol Evol. 2014 May 13;4(12):2352–9. doi: 10.1002/ece3.1095 (PMC4203284; doi:10.1002/ece3.1095)

**Proffitt & Travis. On-line Appendix Information**

We used human population size in a region, and numbers of houses, and numbers of boats, but these were highly correlated and gave similar SEM results. Therefore, we chose to use human population size as a reflection of human impact on estuarine systems.

We tested the full model (independent variables: Tree Size, Outcrossing, Mutation, Reproduction, latitude, and HumanPop); a model with tree size (and all its paths) removed; a model with human population removed; and a model with both tree size and human population removed. Table S1. Shows the comparison of these models for model probability calculated from AIC.

Table S1. The four SEM models tested. K=number of manifest variables in the model. Model probability of the full model was 0.64; and was about twice as likely as the next best model.

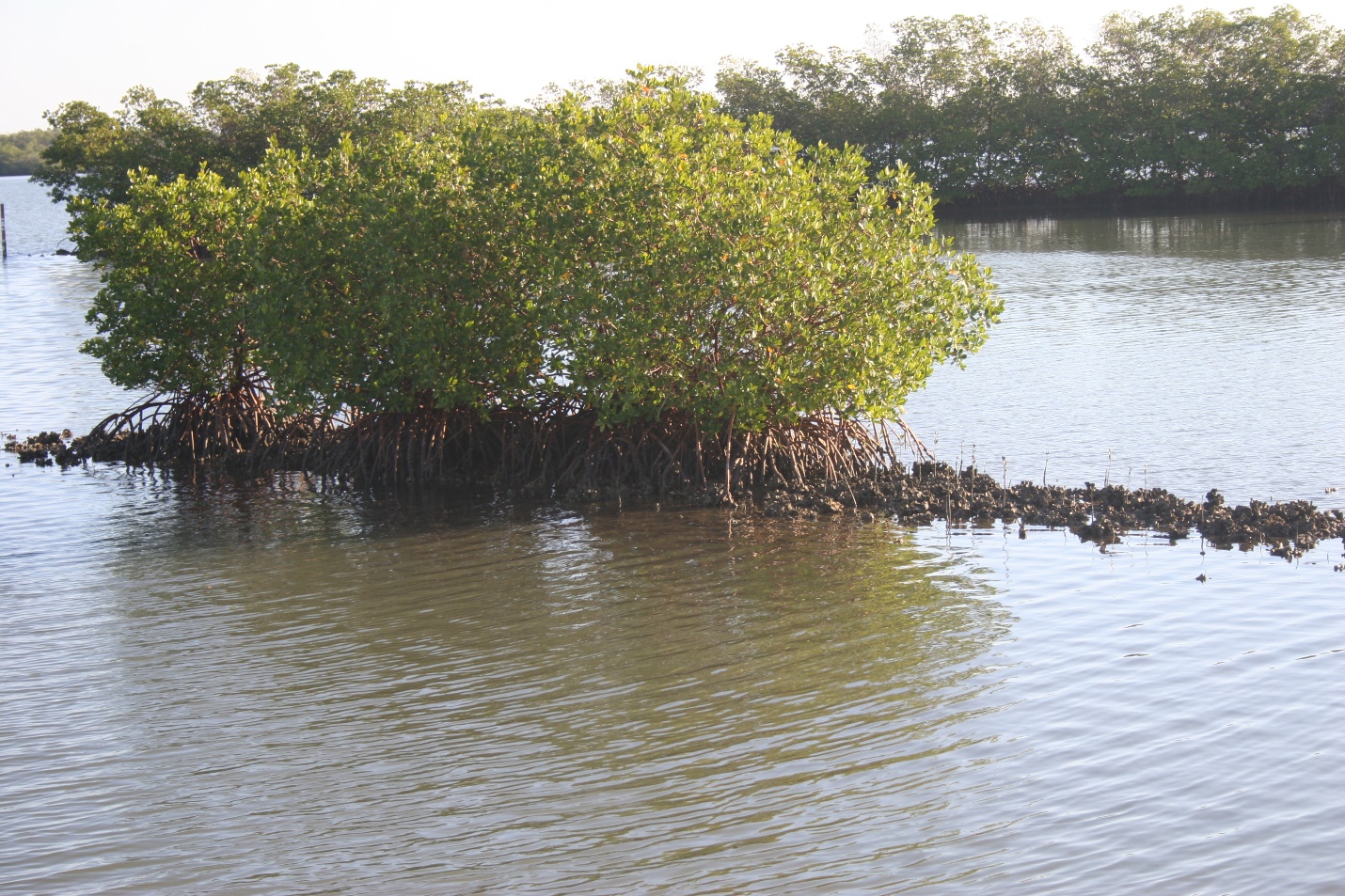

Supplement: Supplementary file 1 — Table S1. The four SEM models tested. [file ece30004-2352-sd1.docx]
